# Large-Area Flexible Photopolymerized Scaffolds: Fabrication and Application to Cardiomyocytes

**DOI:** 10.1021/acsami.5c20678

**Published:** 2026-01-13

**Authors:** Nazar Farid, Sogol Kianersi, Ayesha Sharif, Andrew C. Daly, M. Çağatay Karakan, Christopher S. Chen, Gerard M O’Connor

**Affiliations:** † NCLA Laser Laboratory, Physics, School of Natural Sciences, 8799 University of Galway, Galway H91 TK33, Ireland; § Biomedical Engineering, School of Engineering, College of Science and Engineering, 8799 University of Galway, Galway H91 HX31, Ireland; ‡ CÚRAM, Research Ireland Centre for Medical Devices, University of Galway, Galway H91 W2TY, Ireland; ∥ Department of Biomedical Engineering and the Biological Design Center, 1846 Boston University, Boston, Massachusetts 02215, United States; ⊥ Wyss Institute for Biologically Inspired Engineering, Harvard University, Boston, Massachusetts 02215, United States

**Keywords:** scaffolds, photopolymerization, annealing, laser writing, cardiomyocytes, maturation

## Abstract

Direct laser writing is a remarkable process for digitally sustainable and fully customized manufacturing of medical components. This study reports on the fabrication of large-area 2D polymer-based scaffolds by ultrashort laser pulses. Designs for scaffolds can be fabricated in minutes over large centimeter areas. They are free-standing, thin, and flexible, with feature sizes down to a few microns. Isotropic and non-isotropic fibrous-like geometries are possible. Scaffolds can be rendered electrically conducting in whole or in part. They have excellent deformability; they can be elastically strained to 20% without fracture. Adhesion, proliferation, and alignment of human-induced pluripotent stem cell-derived cardiomyocytes thrive when deployed on scaffolds. Cells are shown to mature well. A comprehensive network of sarcomeres and contractile agility is also observed across the scaffold. Synchronized beating of cells is observed over large areas for time scales of up to 30 days. Evidence of the periodic deformation of the scaffold due to the cyclic forces exerted by the beating cells is observed. The approach is promising for industrial-scale fabrication of 2D scaffold structures tailored to the geometrical, mechanical, and electrical requirements of many cell and tissue targets beyond cardiomyocytes, with future 3D structures realizable by folding or layering.

## Graphical abstract

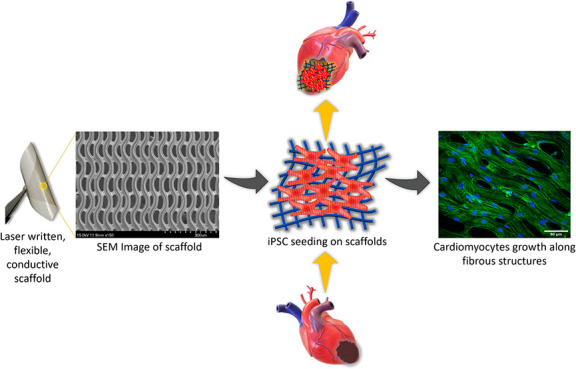

## 1 Introduction

Biofunctional scaffolds are used to localize and organize therapeutic cells by providing mechanical support for their assembly; they can be prepared from either a natural or synthetic material to mimic the extracellular matrix (ECM). Several approaches have been developed to build biomimetic scaffolds. Hydrogel and elastomer materials shaped in the form of fibrous films and cell sheets are the most common structures fabricated by traditional routes such as 3D printing, molding, and electrospinning.^1^ Despite major advances in scaffold engineering, it has often remained difficult to generate structures with the desired microarchitecture, mechanics, and cytocompatibility, etc. Photolithography provides a batch-based approach to prepare scaffolds with the desired precision.^2^ The materials used for conventional photolithography require several processing steps, including etching steps in a harsh environment, the residue from which may impact subsequent regenerative cell studies.^3^ To prepare three-dimensional (3D) structures, new high-throughput techniques need to emerge based on layer-by-layer stacking and self-assembly.^4^ Laser-based techniques, including laser scanning holographic lithography, phase-mask lithography, and fabrication of structures by a scanned focused laser beam, also allow full control by which structures can be fabricated directly in 2D^5^ or 3D.^6^ Photopolymerization is a direct writing technique to fabricate complex micro/nanostructures using different light-sensitive materials. Laser-enabled photopolymerization can be a linear or nonlinear optical process that depends on the absorption of a single photon or the simultaneous absorption of two or more photons in the photosensitive material, respectively.^7^ The method of displacing a single focused laser beam determines the potential to realize centimeter-scale structures efficiently. Scalability is partly addressed by increasing the repetition rate or the number of ultrashort laser pulses provided to the monomer per second. Throughput is also enhanced by increasing the focal volume so that the displacement of the beam over the surface can be extended further between successive pulses while maintaining a uniform exposure of the photoresin. It is important that the beam displacement system can operate at a linear speed, which is compatible with the laser pulse repetition rate and spot size. This is to ensure that the beam can be partly or fully displaced to a new volume in the monomer material with every successive pulse. The integration of a high repetition rate laser, scanning mirror beam delivery systems, and integrated motion stages can be a scalable solution. Such integrated systems can manufacture scaffold templates with custom microscale architectures up to square meter scales.

Typically, these polymer structures are electrically insulating or nonconductive. Active electrical stimulation of electrically conductive scaffolds is interesting in specific cases, as it can promote the alignment and synchronized contractions of cells such as cardiomyocytes (CMs), resulting in the enhanced propagation of action potentials leading to better integration and synchronized beating of cells with the host myocardium.^8^ While electrical stimulation of cells on scaffolds is typically addressed by using a conductive media, the potential to realize conductive scaffolds may create new opportunities. The fabrication of electrically conductive scaffolds typically requires elevated annealing temperatures (>600 °C). These elevated temperatures are necessary to obtain the appropriate crystallization of the metal to achieve the required electronic conductivity. Such elevated temperatures typically destroy micron-sized features in many different polymer scaffolds.

The use of ultrashort laser pulses enables a thin metal layer to be annealed without incurring significant lattice temperatures. When an ultrashort laser pulse interacts with a metal surface, the photon energies are absorbed by the conduction band electrons through photon–electron interactions before the laser pulse terminates. As the electronic heat capacity is typically orders of magnitude lower than the heat capacity of the lattice, less energy is required to place the electronic subsystem into a highly energized state while the lattice remains unaffected. The electronic excitations thus change the electron density distribution, giving rise to modified forces between atoms in the solid.^9^ As a result, the modified interatomic forces create coherent atomic motion, and displacements of atoms can take place on a very short time (subpicosecond) scale. Such nonthermal phase transitions occur without energy transfer from electrons to the lattice.^9^ Thus, it is proposed that laser-induced crystallization occurs due to nonthermal solid-state diffusion of atoms from interstitial to substitutional sites over distances of nanometers. This annealing process with femtosecond laser pulses has been successfully demonstrated and reported for gold thin films with IR, green, and UV laser wavelengths,^10^ indium tin oxide,^11^ amorphous silicon,^12^ and molybdenum thin films^13^ with high melting temperatures, without deteriorating the underlying substrate.^14^ Nonthermal low-fluence ultrashort laser-enabled crystallization is highly relevant for biological materials that cannot tolerate processing temperatures in excess of 200 °C.

A low-temperature sputter-coated process can add a nanometer-thin gold layer on the scaffold structures. These metallic-coated, nonconductive or highly resistive scaffolds are then selectively annealed with ultrashort laser pulses at very low fluences to enhance their electronic conductivity. The value proposition of scaffolds with inherent electronic conductivity is that they are directly addressable and enable more direct coupling of electrical stimulation while maintaining mechanical flexibility. To avoid any change in the mechanical properties, only a thin layer of conductive material should be deposited on the polymer. The addition of a very thin layer does not offer the desired electrical conductivity unless a postdeposition laser annealing step is implemented effectively.

One application of this technology is the development of a scaffold for the heart. Myocardial infarction (MI), resulting from the blockage or insufficient blood supply to the heart, contributes to many deaths every year.^15^ After MI, due to the minimal regenerative capacity of cardiomyocytes in the adult human heart, the damaged myocardium is replaced by fibrotic scar tissue with loss of mechanical pumping function, ultimately leading to heart failure.^16^ A heart transplant is the only decisive curative strategy for patients, but it is available to only a small fraction of the population because of a serious shortage of donors, challenging surgical procedures, and significant associated morbidities.^17^ A repair using an implantable scaffold is another option. The attachment, maturation, and beating of cardiomyocytes are impacted by the mechanical properties of the scaffolds. The stiffness, elastic limit, and mechanical resonant frequencies should mimic the characteristics of the heart’s extracellular matrix (ECM).^18^ Scaffolds should be capable of supporting thousands of cycles of heart contraction and expansion without plastic deformation. The dynamic loading, such as periodic stretching, can reinforce cardiomyocyte alignment and functionality.^19^ The mechanical forces help to enhance the electrical coupling between the cells, which promotes the expression of genes associated with cardiac maturation, calcium handling, and synchronization of heart muscle tissues associated with periodic beating.^20^

In this study, a scalable method to fabricate precision biocompatible scaffolds is investigated. The method is based on a custom laboratory system for photopolymerization. Using a commercial negative-tone photoresin, which becomes less soluble when exposed to laser light, we prepared easily detachable scaffolds. The process produces various geometries with features down to a few microns over large areas up to 50 × 50 mm^2^, on short time scales of only a few minutes. A method is presented to render these scaffolds electrically conductive without sacrificing flexibility. Finally, the interactions with cells are reported. The significance of the study is that it presents a perspective on what can be achieved in the future using scalable laser-based manufacturing tools to enhance regenerative cardiology.

## 2 Materials and Methodology

Figure 1a describes the steps and protocols involved in direct laser writing (DLW) of flexible scaffolds, including the scaffold detachment process.

**Figure 1:**
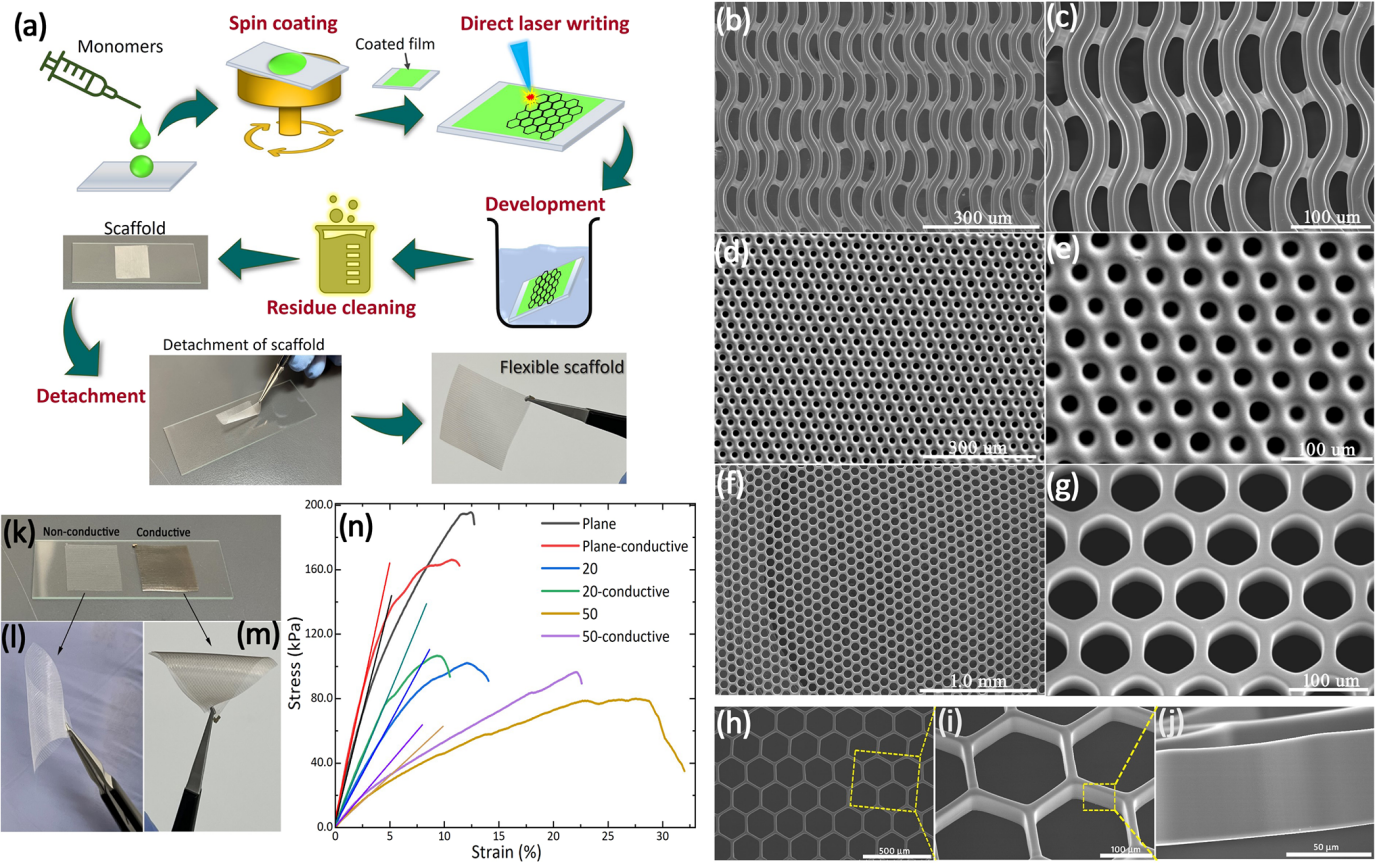
(a) A schematic illustration of the steps and protocols involved in direct laser writing of flexible scaffolds, from preparation to the detachment process. (b–j) Sections of large-area scaffolds prepared by DLW. (b, c) Fibrous-like structures, (d, e) porous structures with 20 μm holes, and (f, g) hexagonal structure scaffolds, respectively. (h) SEM image of larger hexagonal structure scaffolds, while (i) shows the lateral and vertical surface quality at high magnification. (j) Cross-section of the scaffold. (k) Nonconductive and conductive scaffolds prepared from the IP-Visio resins. The bending of (l) nonconductive and (m) conductive scaffolds. (n) Stress–strain behavior of different structures based on conductive and nonconductive scaffolds consisting of a continuous plane sheet, and 20 and 50 μm hexagonal structures. The straight lines show the linear fitting for estimating Young’s modulus.

### 2.1 Preparation of Photoresin

Commercially available acrylic-based photoresins (IP-Visio) from Nanoscribe GmbH were used for laser photopolymerization. While recent studies in the literature indicate that IP-Visio is a good candidate for future implantation applications,^21^ it is proposed here as a representative photopolymerizable scaffold. The monomer was coated on the glass slide by using a spin coater (Laurell WS-400B-6NPR/LITE). The amount of deposited photoresin material was approximately 350 μL. The glass slides were cleaned with acetone and 2-propanol and dried with dry air prior to monomer coating. Spin coating was performed at ambient conditions at 3200 rpm for 20 s. During this process, the photoresin spreads over the glass surface evenly from the center toward the edges under the applied centrifugal force, resulting in a film of uniform thickness. The even nature of the initial film is an important consideration to reduce any errors caused by nonuniform reflection associated with an uneven liquid surface during the laser writing process. The resulting thickness of the coated resin was about 150 μm after the spin coating. During laser writing, the photoresin containing the photoinitiator is excited on absorption of the laser energy, and this subsequently drives the free radical polymerization within the volume where the laser is focused.

### 2.2 Laser Photopolymerization

A 515 nm wavelength laser (second harmonic of 1030 nm) is used for initiating photopolymerization. In this study, direct laser writing was performed using a galvo scanning mirror system (Scanlab) with a femtosecond (500 fs) laser (Amplitude Systems) at a 300 kHz repetition rate. The F-theta focusing lens was used to focus laser pulses into a spot with a radius (ω) of 24 μm. The scanning mirror system provided the advantage of high scanning speeds, even in the writing of curvilinear trajectories, where the F-theta lens provides the flat field of operation, which is essential for high resolution. The glass slide, coated with the photoresin, was placed on a 3D XYZ computer-controlled stage, which provides highly accurate 3D alignment of the writing position under the galvo scanning system. The specific design structures and feature sizes were chosen to fabricate a functional scaffold and to support cell interaction. The design of the scaffold, prepared using computer-aided design, was then transferred via direct machine control software to control the mirror scanning and 3D stages during the laser writing process. On completion of the laser writing step, the scaffolds were placed in propylene glycol methyl ether acetate for development for 20 min, and any residue or unpolymerized resins were then washed away with isopropyl alcohol. In the final step, the prepared scaffold was separated from the glass slide by tweezers, as shown in Figure 1a. The surface morphology was investigated using a digital microscope (Keyence VHX-7000) and a high-resolution scanning electron microscope (Hitachi S-4700). For cell studies, the samples were analyzed using a confocal microscope (Fusion benchtop, DC43, Andor, Oxford Instruments), where the quantification of cells was analyzed with ImageJ.

### 2.3 Preparation of Electrically Conductive Scaffolds

A low-temperature sputter-coated process was used to add a nanometer-thin gold layer to the scaffold structure. A laser annealing process utilizes ultrashort laser pulses at very low fluences to enhance the electrical conductivity of the sputter-coated scaffolds. The scaffolds are first coated on both sides using a sputter coater at room temperature, at a 25 mA deposition current for 2 min. The thickness of the coated gold layer is 12 ± 3 nm, measured using a white light profilometer. The scaffolds are then transferred to a clean room for an established laser crystallization process^10^ using a femtosecond laser. The annealing is performed at a very low fluence of 35 mJ cm^–2^, which is less than the threshold fluence required for the onset of damage. In this way, a carefully controlled laser process is used to (a) enable electronic conductivity and (b) prevent unwanted remelting or damage to the scaffolds.

### 2.4 Preparation of Cells

Two separate hiPSC lines were used to monitor the interactions with the scaffold in this study. Both approaches differentiated human iPSCs into cardiomyocytes. In the first cell line, hiPSCs were purchased from Gibco ThermoFisher Scientific and were cultured on a Matrigel-coated well plate, fed with E8 (STEMCELL Technologies). The hiPSCs were passaged at 85–95% confluency. The differentiation of hiPSCs into cardiomyocytes was carried out using the known GiWi protocol^22^ with a small modification. Briefly, the differentiation was initialized by replacing E8 media with RPMI medium (Gibco, ThermoFisher Scientific) supplemented with B27 without insulin (Gibco, ThermoFisher Scientific) and 8 μM CHIR99021 (STEMCELL Technologies), which is an activator of the Wnt pathway. After 24 h, the media was replaced with RPMI + B27 minus insulin supplemented with 4 μM CHIR99021. On day 3, the Wnt pathway was inhibited by the addition of RPMI + B27 minus insulin supplemented with 5 μM IWP4 (STEMCELL Technologies). The media was replaced with RPMI + B27 minus insulin on day 5. On day 8, a new media, including RPMI + B27 with insulin supplemented (Gibco, ThermoFisher Scientific) was added and refreshed every other day until day 14. Differentiated hiPSCs were maintained in the same culture medium for the experiment.

The second hiPSC line was created from the PGP1 donor from the Personal Genome Project and edited to have an endogenous green fluorescent protein tag on one titin allele (GFP-TTN, a gift from the Seidman Lab at Harvard Medical School).^23^ These hiPSCs were also cultured on a Matrigel-coated well plate, fed with mTeSR1 or mTeSR+ (STEMCELL Technologies), and passaged at 70–90% confluency. The differentiation and maintenance protocol are similar to the above, except 12 μM CHIR99021 was used for 24 h to activate the Wnt pathway, with a media change of RPMI + B27 minus insulin on Day 1. Wnt pathway inhibition is the same as above, and after 11 to 13 days of differentiation initiation, hiPSC-derived cardiomyocytes (hiPSC-CMs) were purified using RPMI, no-glucose media (Thermo Fisher Scientific) with 4 mM Sodium-dl-Lactate solution (Sigma-Aldrich) for 4 days, with the media changed every other day. Following selection, cardiomyocytes were replated onto Matrigel-coated plates and maintained in RPMI 1640 medium supplemented with GlutaMax and B27.

Cell viability of the hiPSC-CMs was investigated using a live/dead assay kit. Briefly, the samples were incubated in 3 mM Calcein AM (Sigma-Aldrich) and 2 mM ethidium homodimer-1 (Sigma-Aldrich) for staining the live and dead cells, respectively (at 37 °C for 1 h). The samples were analyzed using a confocal microscope (Fusion benchtop, DC43, Andor, Oxford Instruments). For quantification, three samples in each group of scaffolds were imaged and analyzed with ImageJ.

For immunofluorescent staining, the samples were fixed in 10% formalin for 1 h at 4 °C in a 24-well plate. Following three times washing with PBS, the fixed samples were incubated in a permeabilizing solution (2% BSA, 0.2% Triton X-100) at room temperature for 30 min. Before staining, the samples were incubated in blocking buffer (2% BSA) for another 1 h. Primary antibodies that were used for staining the samples were antisarcomeric α-actinin (Abcam, ab137346, 1:200) and anticardiac troponin T (Invitrogen, MA5–12960, 1:200). Samples were incubated with primary antibodies at 4 °C overnight. The samples were incubated with secondary antibodies of Rabbit antimouse IgG H&L (Alexa 594, Abcam, ab150116, 1:200) and Goat antirabbit IgG H&L (Alexa 488, Abcam, ab150077, 1:200) for 2 h. The samples were washed three times with PBS, stained with DAPI for 15 min, and images or videos were taken using confocal microscopes (DC43 Fusion benchtop or Dragonfly, Andor, Oxford Instruments; Zeiss Axiovert 200 M inverted spinning disk microscope). For the fiber scaffold, tissue fluorescence images were acquired using the upright confocal multiphoton microscope (Leica TCS SP8MP), operated in single-photon mode, using a 40× water immersion lens.

For the deployment of cells, the scaffolds were sterilized by submerging them in ethanol for 30 min and exposing them to UV light for another 30 min. The scaffolds were coated with Matrigel, placed inside 24-well plates, and kept flat at the bottom of the plate using a PDMS-made ring. 350,000 hiPSC-CMs were cultured on top of each scaffold and kept for further investigations. In the experiment using the PGP cell line with GFP-TTN, PDMS (1:10 weight%, Sylgard 184) cell culture wells were generated using an 8 mm diameter biopsy punch (Integra), and the 10 × 10 mm^2^ scaffolds were placed on top. Subsequently, PDMS reservoirs with the scaffolds were bonded to a glass-bottom Petri dish (Mat-Tek) after plasma-treating both surfaces (Herrick Plasma, 10.5 W RF power, 60 s). This process clamped the scaffold to a glass-bottom Petri dish while allowing the cells to access the bottom of the scaffold. Then, these scaffolds were sterilized using the same procedure and coated with Matrigel (Corning) mixed 1:80 in DMEM/F-12 (Fisher). Before seeding, the scaffolds were washed with PBS, and 250,000 hiPSC-CMs were cultured on each scaffold. The cells were maintained in RPMI 1640 supplemented with GlutaMax and B27 and kept for further investigations.

## 3 Results and Discussion

The laser-enabled polymerization process consists of three phases: initialization, growth, and termination. During the initialization phase, the photoresin within the laser-focused spot absorbs energy to initiate the decomposition of the photoinitiator, which generates free radicals.^24^ In single-photon polymerization, every photon transmitting through the photoresin has a probability of generating radicals. If the incident fluence is higher than a threshold fluence, discernible polymerization will be observed. Hence, the incident laser fluence, the Rayleigh range (or depth of focus), combined with the cross-sectional area of the incident laser beam, determines the precision of the polymerization process. If the thickness of the photoresin is less than the Rayleigh range, then it is the thickness of the monomer layer that determines the depth over which the polymerization takes place. Multiphoton polymerization involves the simultaneous absorption of two or more photons. In the case of two-photon polymerization, optical absorption is proportional to the intensity squared times the number of photoinitiator molecules in the cross-section of the incident laser beam. The square of the intensity causes an increased effective localization of the excitation within the focal volume and results in structures of higher precision.^25^

A high numerical aperture (NA) focusing lens is often used to minimize the irradiated volume. It is typical for multiphoton polymerization with high NA optics to produce a striated or layered structure due to the variation of the laser fluence in the direction of travel of the light beam; each striation is indicative of the actual precision over which the photopolymerization takes place within the focal volume. Maintaining the nonpolymerized boundary at the interface of the photoresin and the substrate ensures that the polymerized structure can be easily released, as it is not adhered to any host material. The growth phase begins after optical absorption and free radical formation. In this phase, the free radicals bond to monomer chains, forming larger free radical molecules. This reaction further propagates through the radicalization of other monomers within a volume that marginally extends beyond the region that is irradiated. The confinement of polymerization to the irradiated volume is an important consideration in terms of the length of the chains that are produced. This, in turn, impacts the mechanical properties, specifically their flexibility. The third phase of polymerization terminates the process by combining long-chain free radicals with other radicals or initiators, thus ceasing further reactions leading to polymerization.^24^

### 3.1 Scaffold Structure

Figure 1(b–j) presents SEM images of different structures of the laser-written scaffolds. A laser fluence of 195 mJ cm^–2^ initiated the direct writing of the structures. The structures are large areas extending continuously over 50 × 50 mm^2^. Three types of structures are presented here. Figure 1(b, c) shows vertical fiber-like structures held together with horizontal cross-links. Figure 1(d, e) shows porous structures defined by 20 μm diameter holes, and Figure 1(f, g) shows hexagonal structures defined by a 20 μm length of each side, respectively. The polymerization of the acrylic-based monomer was achieved using a laser scanning speed of 200 mms^–1^, along a single line trajectory, which resulted in 48 laser shots (or pulses) per focused laser spot, delivered to each section of the polymerized scaffold. This results in a high pulse-to-pulse spatial overlapping of 96%. Additional high-resolution SEM images in Figure 1(h–j) provide a detailed side-on evaluation of the scaffold structures. Figure 1(i) shows an excellent surface finish of the edge of the scaffold structures. The cross-sectional view in Figure 1(j) confirms that there are no striated or stacking layers; surfaces are free from any observable defects. The width of the structures is 10 μm with a height of 120 μm, resulting in a depth-to-width aspect ratio of 12, respectively.

The laser intensity had a Gaussian distribution across the 48 μm spot within the focused beam. Monomers are transparent to the laser at intensity values below the absorption threshold intensity.^26^ This means that monomers do not absorb the incident laser in the low-intensity regions and only absorb the incident radiation at the very center of the Gaussian beam, where the intensity or the square of the laser intensity exceeds the threshold required for the onset of photoinitiation. This dependence of absorption on the incident beam profile helps to attain the high spatial resolution for structures presented in Figure 1. By careful control of the incident fluence, a lateral resolution of approximately 5 μm is attained with a laser spot diameter of 48 μm using a single laser scan.

The precision and edge quality of the structures are excellent, particularly given that a galvo scanning beam delivery system was used. The question that arises is whether the photopolymerization process is based on a single-photon or two-photon process. The energy carried by a single photon at the recommended wavelength of 780 nm for the photoresin is 1.59 eV. A total energy of 3.18 eV is required for two-photon polymerization at the 780 nm laser wavelength. The photon energy for the 515 nm wavelength is 2.41 eV, which is 0.82 eV less than required for the two-photon threshold at the 780 nm wavelength. A total energy of 4.82 eV will be absorbed by the photoinitiator for two-photon polymerization at the 515 nm wavelength, which is 1.64 eV higher than the energy required in two-photon polymerization by the 780 nm laser wavelength. While a further detailed spectroscopic study may be required to confirm, it is most likely that a two-photon process is at play at 515 nm, with the excess energy going to create heat, resulting in the smooth morphology of the scaffold structure, which is an important design parameter for scaffolds.

### 3.2 Conductive Scaffolds

The laser-enabled conductivity confirms that highly conductive samples were established with a resistance that is less than 10% of the bulk values. Typical resistance values of 1 Ω are measured across a 20 × 20 mm^2^ scaffold. Figure 1(k) presents an image of the conductive and nonconductive scaffolds. Prior to laser-induced crystallization, the layer was not electrically conductive. Post-laser treatment, the sheet resistance determined by a 4-point probe (Ossila) is 27 ± 2.0 × 10^–2^ Ω□^–1^. The corresponding resistivity is estimated to be 2.7 × 10^–7^ ± 2.0 × 10^–10^ Ωm. The electrical conductivity is given by 3.70 × 10^6^ ± 2.76 × 10^3^ Sm^–1^. The microstructural transformation of the thin gold layer arising from the laser annealing step is not observable using the techniques available in this study.

### 3.3 Mechanical Properties

The conductive and nonconductive scaffolds both exhibit significant flexibility with excellent compression and bending; the detachable scaffolds can be rolled or folded with extremely small radii of curvature down to a 3 mm radius of curvature (Figure 1(l and m)). A video demonstration is presented in the Supporting Information showing how three-dimensional (3D) scaffolds can be produced from 2D by folding or rolling (Movie S1 and Movie S2). This shape-recovering property and flexibility convey the potential for deploying similar tissue scaffolds using a minimally invasive procedure in future.^27^

The mechanical properties of conductive and nonconductive scaffolds were analyzed quantitatively using a universal tensile testing machine (Zwick Uniaxial). The stress–strain curves of plain polymerized sheets, scaffolds consisting of 20 μm hexagonal and 50 μm hexagonal cells are shown in Figure 1(n). The measured elastic modulus and yield strength are summarized in Table 1. Mechanical measurements of the scaffold confirm that the effective elastic modulus of the scaffolds can be tuned by the geometric structure that is written into the scaffold. Anisotropic structures consisting of elongated fibers give rise to anisotropic mechanical properties as well. The deformation of the scaffold is both linear and nonlinear. Elastic deformations are observed up to 20% strain.

**Table 1:** Elastic Modulus and Yield Strength of Conductive and Nonconductive Scaffolds

**Scaffolds type**	**Elastic modulus (kPa)**		**Yield strength (kPa)**	
	***Nonconductive***	***Conductive***	***Nonconductive***	***Conductive***
50 μm hexagonal structure	629 ± 21	793 ± 57	94 ± 0.82	81 ± 0.78
20 μm hexagonal structure	1252 ± 42	1635 ± 64	79 ± 0.20	84 ± 1.10
Continuous plane sheet	2727 ± 67	3176 ± 19	195 ± 0.15	162 ± 0.95

Cardiomyocytes are very sensitive to the mechanical properties of scaffolds. For example, stiffer scaffolds may inhibit cell elongation and disturb the sarcomere development, while softer scaffolds may lead to an immature phenotype.^28^ In other words, the type of mechanical load experienced by the cardiomyocytes defines their physiological or pathological status.^29^ The elastic moduli presented in Table 1 are less than the values typically obtained for IP-Visio. It is also significant that the larger the porous structure, the smaller the elastic moduli. The mechanical results confirm that the scaffolds are stiffer than the typical natural environment for cardiomyocytes; however, the larger structures of the scaffolds enable them to distort by bending. It should be noted that mechanical measurements were performed just after preparation, before the scaffolds became dry. The dry and wet status of scaffolds has a significant effect on the mechanical properties, as IP-Visio has been shown to reduce its Young’s Modulus up to 3-fold in wet conditions.^30^ This dependence on wetness is challenging to replicate during tensile measurements, as the scaffold dries and becomes stiff. Samples can often break at the point of clamping in the tensile tester. The Young’s Modulus values (Table 1) attained from our scaffolds are relatively better than those previously reported for IP-Visio in multiphoton polymerization.^30,31^ This difference could be due to the hexagonal geometric structure produced in our scaffolds.

### 3.4 Interaction of Cells with Laser-Written Scaffolds

As presented in Figure 2, both the qualification and quantification results of the live/dead assay demonstrate no significant difference between gold-coated and noncoated scaffolds in each group between Day 1 and Day 7. The scaffolds did not negatively affect the hiPSC-CMs’ viability. Also, as shown in Movie S3, the early-stage hiPSC-CMs retained their contractile functionality after being cultured on the scaffolds for 7 days. The immunohistochemical detection of cardiac proteins was performed to further examine the interaction of hiPSC-CMs with the scaffolds. The hiPSC-CMs that were cultured on top of the scaffolds kept expressing the cardiac markers of cardiac troponin (cTnT) and the sarcomere marker α-actinin (Figure 2c). As can be seen in the images, the cells were oriented in the same alignment as the principal axes of the scaffold structure.

**Figure 2:**
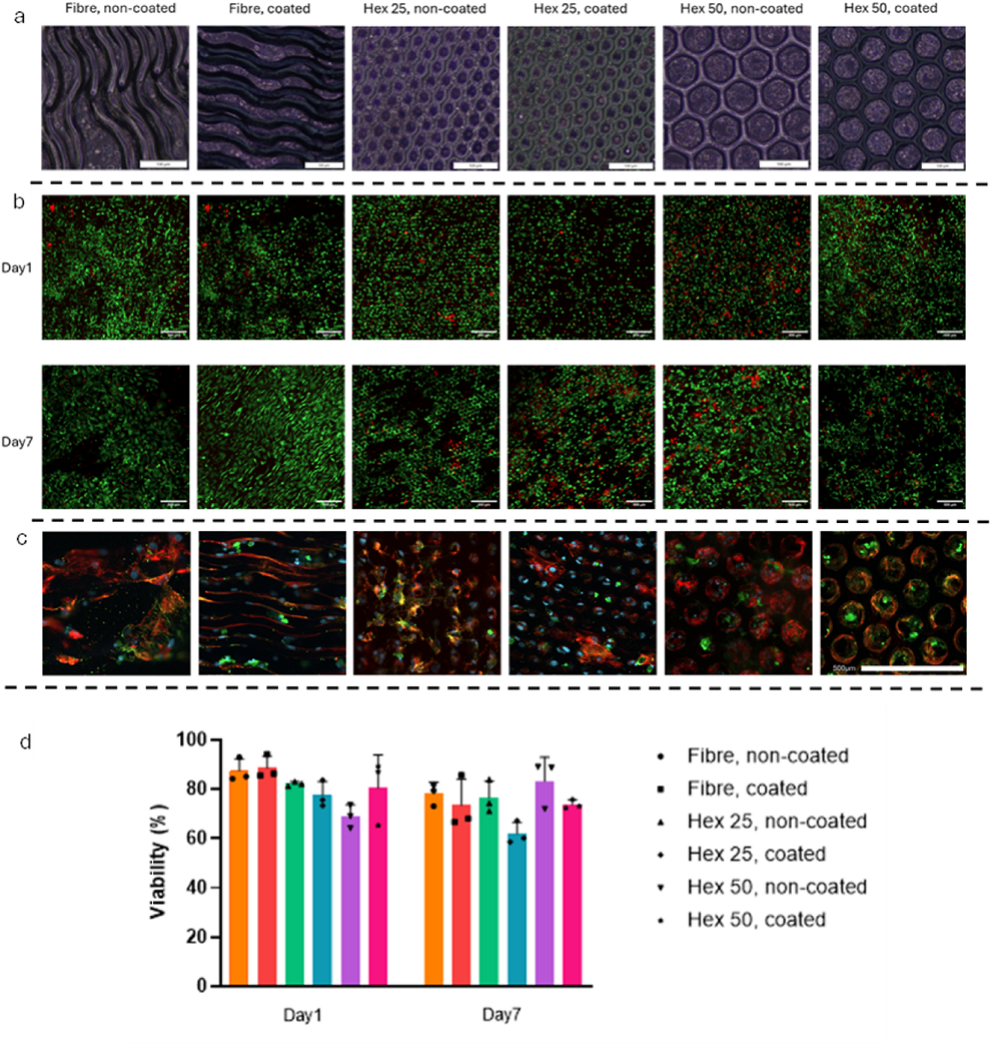
(a) Depicts the light microscopy images of the first cell line consisting of hiPSC-CMs cultured on the surface of different groups of scaffolds (scalebar = 100 μm). (b) and (d) Demonstrate the live/dead images (scalebar = 200 μm) and quantified results of the live/dead assay, respectively, for each group of scaffolds on Day 1 and Day 7. The results show no significant difference in the viability of hiPSC-CMs cultured on scaffolds between Day 1 and Day 7 (one-way ANOVA, *n* = 3). (c) Illustrates the expression of cardiac markers cTnT (red) and α-actinin (green) in hiPSC-CMs on top of the different groups of scaffolds (scalebar = 500 μm).

The second cell line, consisting of the TTN-GFP-expressing cells seeded on the scaffolds, was also functional and synchronously beating on both coated and noncoated scaffolds over a longer period of more than 31 days (Movie S4). An open-source software (MUSCLEMOTION)^32^ was used to visualize the collective motion of these sarcomeres, which determines dynamic changes in pixel intensity between image frames and reports a relative measure of movement during muscle contraction and relaxation. This method visualized synchronous contractions with clearly defined peaks resulting from the pixel intensity changes from the entire 0.8 × 0.8 mm^2^ field of view attained with the objective (Movie S5), after a week (Figure 3a) and a month (Figure 3b) after seeding, for both noncoated and coated (Figure 3c) scaffolds.

**Figure 3:**
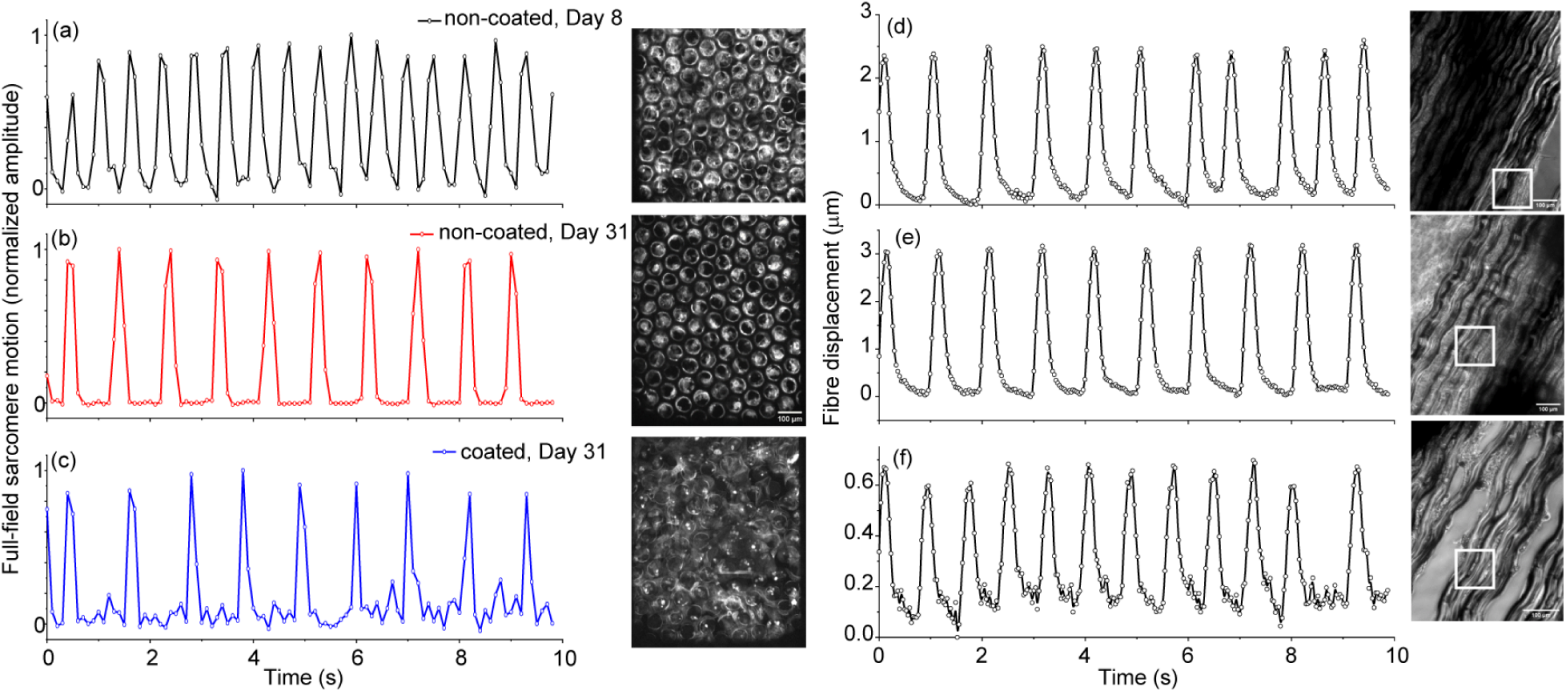
Synchronous contractions of the sarcomeres on noncoated (a: Day 8 after seeding, b: Day 31 after seeding) and coated Hex 50 (c) scaffolds, over about 0.8 × 0.8 mm^2^ regions, are shown on the right. (d–f) Displacement of fibers due to the spontaneous contractions of hiPSC-CMs, taken from three different regions of the scaffold. Representative regions of interest correspond to rectangular regions in the images and their counterparts in Movie S7. Scale bars are 100 μm.

Although the low frame rates did not allow for quantitative comparisons between the contractile dynamics of noncoated and coated scaffolds, sarcomeres on the gold-coated scaffolds typically had a smaller signal-to-noise ratio, which could be due to cell expression and/or the reflective properties of gold when viewed with the inverted spinning disk microscope (Zeiss Axiovert 200M).

Regarding the potential use of these scaffolds as a cardiac patch, the scaffolds should be deformable by the beating of cardiomyocytes to improve the pumping function of the heart ventricle. To decrease the stiffness imposed on the scaffold by the boundary conditions and potentially see the periodic displacement of the scaffold, we suspended the fibrous scaffold between two PDMS rings, then seeded 90% hiPSC-CMs and 10% human cardiac fibroblasts (Lonza) in a collagen-based hydrogel, using the protocol described in ref. 33 for cell seeding and displacement quantification. Over the course of 10 days, the cells integrated with the scaffold and began to displace the fibrous scaffold on a scale of a few microns, as shown in Figure 3(c–e) and Movie S6

Green fluorescent titin protein-expressing iPSC-CMs seeded on scaffolds were investigated in more detail for their interactions with both noncoated and coated scaffolds. Movie S7 and Movie S8 are representative videos that show stacks of images taken from noncoated and coated hexagonal scaffolds with a 50 μm side length (Hex 50), respectively, that correspond to a height of about 68 μm, starting from a few micrometers below the bottom surface of the scaffolds. Projections of these images are shown in Figure 4 (a: Hex 50 noncoated, b: Hex 50 coated). These images show a clear infiltration of cells into the scaffolds through hexagonal pores, as well as some interaction between the cells within the pores. For coated fiber scaffolds, we used an upright microscope to image the cells with less interference from the conductive coating on the scaffold. Here, we also observed that the hiPSC-CMs adhered to the fibers and forming connections between the gaps (Figure 4c). In all scaffolds, a closer examination of titin expression revealed that the associated *Z*-disks of the cells are pronounced and striated on the scaffolds. Although the isotropic nature of the hexagonal geometry prevents global sarcomere alignment in a particular direction, we observed some local alignment of the sarcomeres in the direction between neighboring pores (Figure 4a). Upon visual inspection, we observed that *Z*-disks and the corresponding sarcomeres on noncoated scaffolds are typically larger than those formed on gold-coated scaffolds (Figure 4b). In the fibrous scaffold, we observed some alignment of the sarcomeres in the direction of fiber orientation (Figure 4c, diagonal orientation), which suggests that fibrous designs could be a useful strategy in mimicking the aligned extracellular matrix fibers the cardiomyocytes experience in vivo. The length of the sarcomeres between the *Z*-disks of hiPSC-CMs cultured on scaffolds appears to be similar, ranging from 1.7 to 1.9 μm, which aligns well with recent literature regarding hiPSC-CMs (Figure 4, bottom row).^34^

**Figure 4:**
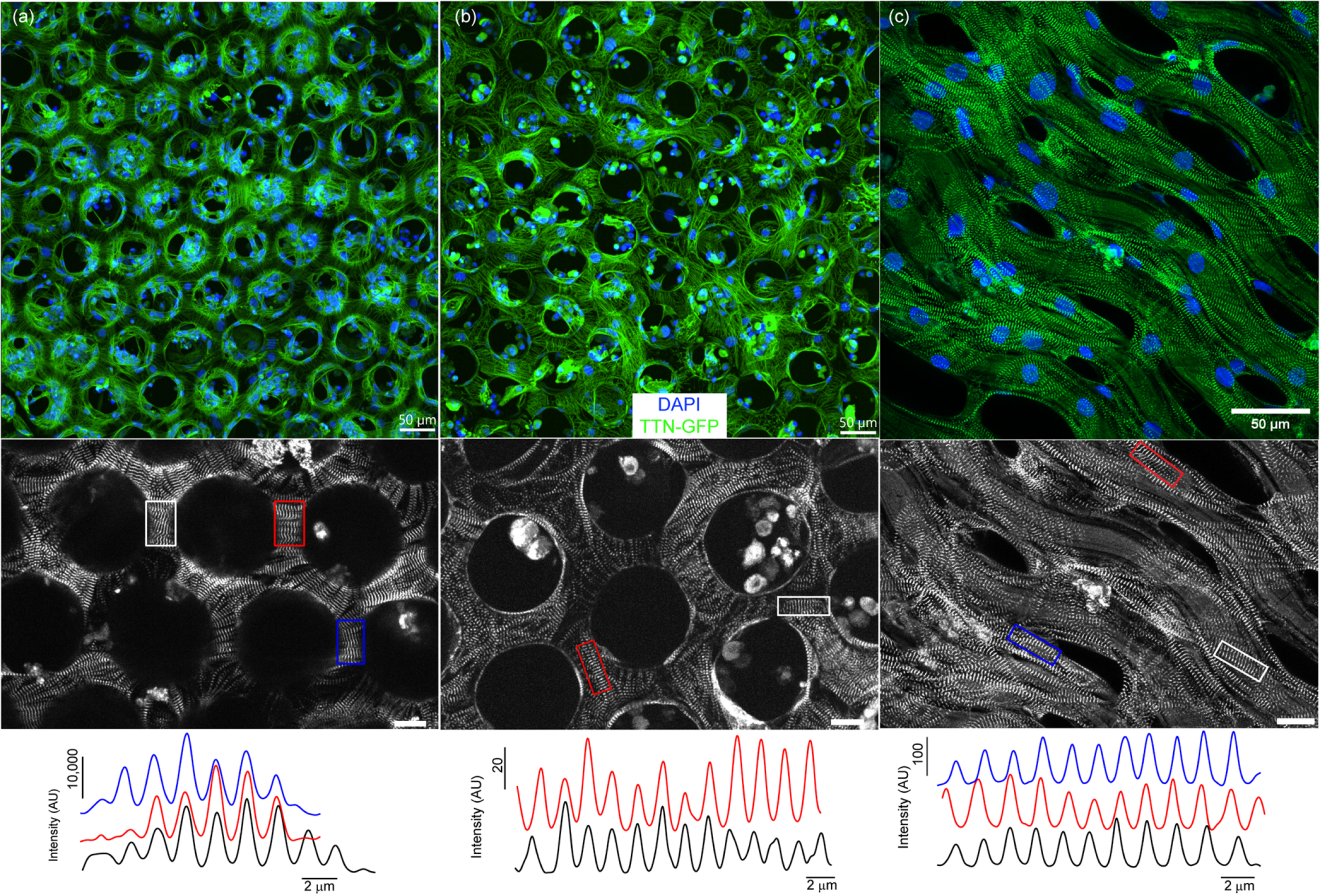
DNA (blue) staining titin-expressing (green) cells occupying (a) Hex 50 noncoated, (b) Hex 50-coated, and (c) fiber-coated scaffolds. Images in the middle are representative images focusing on *Z*-discs and sarcomeres that are directly on the scaffolds (left: Hex 50 noncoated, center: Hex 50-coated, right: fiber-coated, scale bars are 20 μm). Bottom plots show representative intensity profiles taken from regions of interest (left: Hex 50 noncoated, center: Hex 50-coated, right: fiber-coated), showing sarcomere lengths between 1.6 and 1.9 μm.

## 4 Conclusions

This paper presents new perspectives on how large-area flexible scaffolds can be prepared using processes based on an ultrashort pulsed laser. Acrylic-based monomers are easy to process; they are biocompatible and have good mechanical properties. Acrylic resins are also ideal for cell interactions in regenerative medicine and therapies. Their solubility in ethanol is favorable for removing and washing away the unused monomers that are toxic to the cells. Moreover, acrylic structures resist swelling during the development step and, therefore, exhibit low shrinkage. Two-dimensional polymer-based scaffolds, with the ability to be easily detached from the host substrates, are prepared by a direct laser writing process using ultrashort laser pulses. The process can produce various geometries with features down to a few microns in size over large areas up to 50 × 50 mm^2^ within minutes. These highly flexible scaffolds can be easily detached from a host substrate. The prepared scaffolds demonstrate tunable mechanical properties. The mechanical properties of scaffolds depict that the development of different reconfigurable structures can be tailored for various tissue engineering applications. The geometry of scaffold structures can be modified to lead to cell and myofibril alignment. To enable potential electrical stimulation of cells, a low-temperature ultrashort laser-based approach is used to produce conductive scaffolds. Preliminary interaction with cardiomyocytes with scaffolds having different geometrical shapes and conductive and nonconductive surfaces was investigated, showing promising initial results. It was confirmed that the scaffolds were biocompatible and nonharming to cells. In conclusion, the results of the current study indicate that such large-area scaffolds are promising and may realize potential uses in future cell maturation, cell studies, and therapies. Moreover, it is envisaged that 3D structures can be obtained by using rolled or layered 2D structures fabricated as described.

## Supplementary Material



















## Data Availability

The data that support the findings of this study are available from the corresponding author upon reasonable request.

## Notes

Flexibility of uncoated nonconductive scaffold (MOV)
Flexibility of gold-coated conductive scaffold (MOV)
Cell contraction (AVI)
GFP-Titin expressing hiPSC-CMs on 50 μm hex, noncoated, coated, and coated fiber spontaneously beating on scaffolds (MP4)
GFP-Titin expressing hiPSC-CMs on 50 μm hex, noncoated scaffold, spontaneously and synchronized beating across a 0.8 × 0.8 mm^2^ area (MP4)
Phase contrast video of cells spontaneously beating and deflecting the fibers (MP4)
A *Z*-stack of the cells fixed on 50 μm hex noncoated scaffold (MP4)
A *Z*-stack of the cells fixed on coated scaffold (MP4)
hiPSC cells with TTN-GFP on gold-coated hex 25 and on fiber scaffolds (Figure S1) (PDF)
