# Supplementary material for: Large-Area Flexible Photopolymerized Scaffolds: Fabrication and Application to Cardiomyocytes
Source: ACS Appl Mater Interfaces. 2026 Jan 13;18(3):4830–8. doi: 10.1021/acsami.5c20678 (PMC12862759; doi:10.1021/acsami.5c20678)
Supplement: Supplementary file 9 [file am5c20678_si_009.pdf]

Supporting Information

Large area flexible photopolymerized scaffolds: Fabrication and application to cardiomyocytes

Nazar Farid[1,3]*, Sogol Kianersi[2,3], Ayesha Sharif[1], Andrew C. Daly[2,3], M. Çağatay Karakan[4,5], Christopher S. Chen[4,5] and Gerard M O'Connor[1,3]

[1]NCLA Laser Laboratory, Physics, School of Natural Sciences, University of Galway, Galway H91 TK33, Ireland
[2]Biomedical Engineering, School of Engineering, College of Science and Engineering, University of Galway, Galway H91 HX31, Ireland
[3]CÚRAM, Research Ireland Centre for Medical Devices, University of Galway, Galway H91 W2TY, Ireland
[4]Department of Biomedical Engineering and the Biological Design Center, Boston University, Boston, MA 02215, Massachusetts, USA
[5]Wyss Institute for Biologically Inspired Engineering, Harvard University, Boston, MA 02215, Massachusetts, USA

**Corresponding author: nazar.farid@universityofgalway.ie*

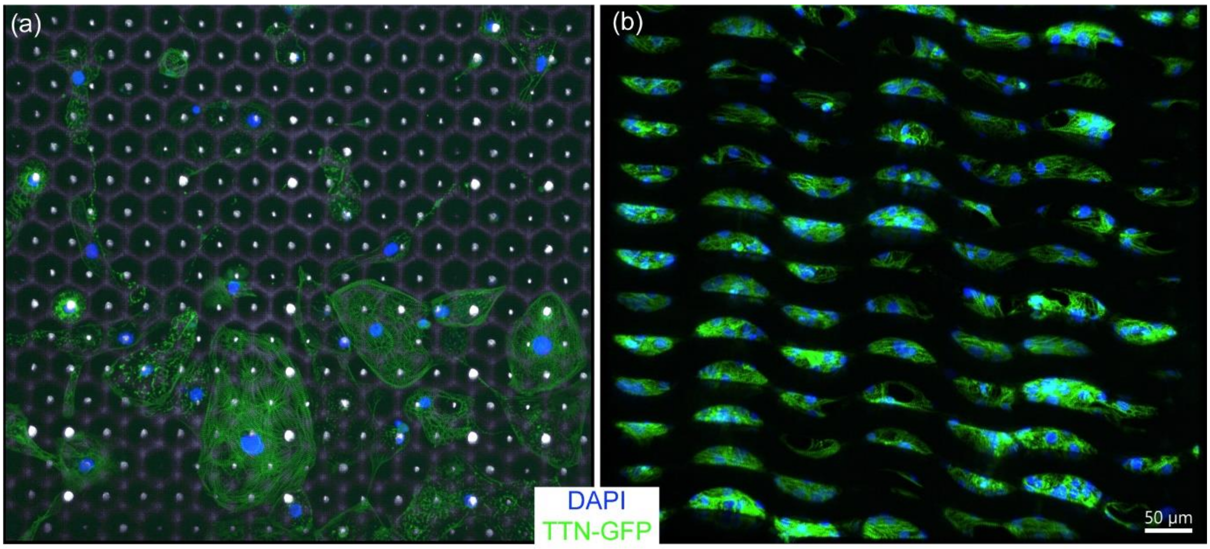

Figure 1. hiPSC cells with TTN-GFP on gold coated Hex 25 (a) and fibre (b) scaffolds. Imaging the cells through to bottom to top was more difficult due to the higher density of material on the scaffold. Smaller openings in 25 µm hexagonal pores also resulted in less infiltration of cells.

Movie S1: Demonstration of flexibility of uncoated nonconductive scaffold.

Movie S2: Demonstration of flexibility of gold coated conductive scaffold.

Movie S3: Day 7, cell contraction.2

Movie S4: GFP-Titin expressing hiPSC-CMs on 50 µm hex, non-coated (left); 50 µm hex, coated (middle) and; Fibre, coated (right) spontaneously beating on scaffolds (Day 31).

Movie S5: GFP-Titin expressing hiPSC-CMs on 50 µm hex, non-coated scaffold, spontaneously and synchronised beating across 0.8 x 0.8 mm^2 area.

Movie S6: Phase contrast video of cells spontaneously beating and deflecting the fibres, taken from three different regions of the scaffolds.

Movie S7: A Z-stack of the cells fixed on 50 µm hex non-coated scaffold, images start from the bottom of the scaffold to about 68 µm up. Blue: nuclei, Green: (Titin).

Movie S8: A Z-stack of the cells fixed on 50 µm hex, coated scaffold, images start from the bottom of the scaffold to about 68 µm up. Blue: nuclei, Green: Titin.
